# Plasma-mediated radiofrequency ablation followed by percutaneous cementoplasty under fluoro-CT guidance: a case report

**DOI:** 10.4076/1757-1626-2-8548

**Published:** 2009-08-17

**Authors:** Gianpaolo Carrafiello, Domenico Laganà, Andrea Ianniello, Federico Fontana, Monica Mangini, Lucia Mocciardini, Emanuela Spanò, Filippo Piacentino, Salvatore Cuffari, Carlo Fugazzola

**Affiliations:** 1 Department of Radiology, University Hospital of InsubriaViale Borri 57, 21100 VareseItaly; 2 Service of Anaesthesiology and Palliative Care, University Hospital of InsubriaViale Borri 57, 21100 VareseItaly

## Abstract

We report a case of a 81-year-old Caucasian man with colorectal carcinoma, treated by surgery in 1998, referred for palliative treatment of a refractory painful caused by osteolytic metastases of 2.5 cm in back-upper ilium spine. Plasma-mediated radiofrequency ablation was performed under conscious sedation, using Fluoroscopic Computer Tomography guidance. After completing the ablation phase of the procedure, a mixture of bone cement and Biotrace sterile barium sulfate was injected into the ablated cavity.

Patient was evaluated by using the Brief Pain Inventory and considering pain interference with daily living at day 1 and 3 and week 1, 2, 3, 4 by means of a telephone interview. A post-procedure Computer Tomography scan was performed to examine the distribution of cement deposition few minutes after the procedure. The plasma mediated RFA and cementoplasty were well tolerated by the patient who did not develop any complication.

## Introduction

Pain management in terminally ill patients with metastases involving bone can be challenging. Conventional therapeutic options for pain control include: radiation therapy (RT), chemotherapy, surgery and the use of analgesic drugs [1].

Despite these measures, the quality of life for these patients is often poor because of intolerable pain. There are many reasons for failure of traditional therapies to control pain: (a) radiation insensitivity of the neoplasm or limitations of radiation dose to normal structures, (b) poor therapeutic response or toxicity of the chemotherapeutic agent, (c) contraindication of major surgery in patients with advanced disease and poor functional status and (d) intolerable analgesic-related side effects that may develop increasing of analgesic doses [2].

Investigators have so explored several alternative strategies for the treatment of painful metastatic disease; these involve the use of percutaneous image-guided methods to locate tissue ablative devices into focal metastatic lesions: ethanol [3], laser-induced interstitial thermotherapy [4], percutaneous radiofrequency ablation (RFA) [5], and, most recently, cryoablation [6].

This study investigates clinical viability of a novel technique for the treatment of painful bone metastases: the plasma-mediated RFA assisted with cement injection.

The plasma-mediated RFA was choice instead of simple RFA because: the exterior margin of the cortical bone was interrupted and this may result in a leakage of cement in the nearby tissues and because the non plasma-mediated RFA sometimes can give a coagulative necrosis which doesn't permit a correct allocation of the cement inside the cavity.

## Case presentation

**Figure 1.**
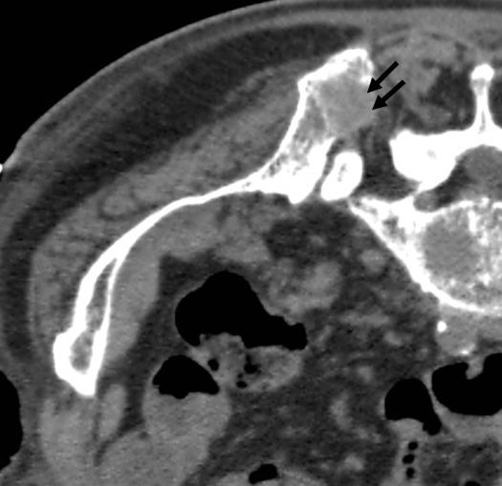
CT scan before the radiofrequency session. CT scan acquired with the patient in a prone position shows an osteolytic metastasis of 2.5 cm in the right back-upper ileum spine (black arrows).

We present a case of 81-year-old Caucasian man with a history of colorectal carcinoma treated by surgery in 1998 was referred for palliative treatment of a refractory painful, osteolytic metastasis of 2.5 cm in back-upper ileum spine, as showed in the Computer Tomography (CT) scans performed before the treatment (Figure 1).

Prior to treatment, patient was assessed by Brief Pain Inventory (BPI), visual-analogue scale (VAS) and recording the use of analgesic drugs.

Before the RFA, the daily therapy of our patient consisted in the following analgesics: 60 mg of morphine, 20 mg of ketorolac-tromethamine and 5 mg of haloperidol; furthermore, he used morphine, at most 30 mg/day, and paracetamol 500 mg, at most 2 g/day.

In the BPI, patient is asked to rate his worst pain in 24 hours, least pain in 24 hours, and average pain, with allowed responses ranging from 0 to 10. Relief of pain through the use of analgesic medications is scored on a scale of 0% (no relief) to 100% (complete relief). Pain interference with daily living is evaluate with questions concerning general activity, mood, walking ability, normal work, relations with other people, sleep, and enjoyment of life, also on a 0-10 scale (0 = no interference, 10 = completely interferes). The patient was asked to answer these questions with respect to the lesion that was to be treated.

Before RFA an imaging-guided biopsy was performed and the frozen specimen sent to Anatomy Patology Laboratory to obtain confirm of malignancy.

An informed written consent was obtained for the patient.

A complete blood count (platelet: 167.000/mm^3^) and prothrombin time (INR: 1,06) were obtained within 24 hours of the procedures.

The patient was treated with prophylactic antibiotics therapy consisted of cefazolin, 1 g, three days before the procedure and repeated every 8 hours over a 72-hours period. The sedation was done in according to the principles of “monitored anaesthesia care” receiving: propofol (90 mg), alfentanyl (0.5 mg) and midazolam (3 mg). The patient received oxygen during the procedures and continuous monitoring of heart rate, electrocardiographic tracing, oxygen saturation, and respiratory rate were obtained, and blood pressure was determined every 4 minutes. Patient skin entry site of the planned needle puncture was anaesthetized with 2% carbocaine. The procedure was performed under fluoro-CT guidance (Aquilion 64/Toshiba/Tokyo/Japan) with the patient in the prone position. Access to the ilium spine was gained by using a 11 G bone marrow harvesting needle that was placed in the back-upper iliac spine to made a biopsy. The bipolar radio-frequency-based device (CAVITY; ArthroCare, Sunnivale, Calif, USA) was advanced into the delivery port (harvesting needle or introducer kit) until its tip protruded beyond the cannula tip. The needle tip was placed with different orientations to obtain a cavity; ablation was ceased when a noticeable reduction in tactical resistance was detected. When inserting the device towards the anterior aspect of the ileum spine, the tissue dissolution setting was used. A total of 4 passes were made to complete the cavity; it was left in place while preparing the bone cement for insertion. After completing the ablation, a mixture of bone cement (Zimmer, Warsaw, Ind, USA) and Biotrace sterile barium sulfate (Bryan, Worburn, Mass, USA) was injected into the ablated cavity under CT-fluoroscopic guidance. On average, 3 ml of bone cement was found to be sufficient to fill the ablated lesion in the back-upper ilium spine. A post-procedure CT scan was performed to examine the distribution of cement deposition few minutes after the procedure. Patient was evaluated for severity of pain by using the BPI and considering pain interference with daily living at day 1 and 3 and week 1, 2, 3, 4 by means of a telephone interview.

The needle was correctly deployed inside the lesion, and the total ablation time was of about 10 minutes. The procedure was considered efficacious and no operative or post-operative complications were observed. The patient experienced a decrease in worst pain over the course of the follow-up period; on the bases of BPI, at day 1 is worst pain had score of 4 in the least 24 hours and the interference of pain with daily living had score of 5. Further, before plasma-mediated RFA, morphine was reduced to 15 mg/day; worst pain remained stable up to week 1. Length of stay in the hospital was 3 days, included the day of the treatment.

## Discussion

Approximately 40% of patients with cancer develop metastatic disease; of these patients, 50% have poorly controlled pain. Achieving adequate pain control is often difficult, and quality of life for the patients is poor [2]. RT is considered the standard care for management of painful bony metastases [7]. Other therapies, including chemotherapy, hormonal therapy, radiopharmaceuticals and surgery, may be used in an attempt to provide palliative pain relief. With these therapies pain relief, when achieved, may not occur until 4-12 weeks after the initiation of the treatment. When these methods are not possible or are not effective, analgesic medications remain as the only current alternative therapy [5].

Although RT is generally considered effective for metastatic bone cancer, this therapy may be administered a single dose; however, most RT schedules involve daily treatments over 2-3 weeks.

The time required to derive maximal benefit following RT for solitary and multiple metastases is usually 12-20 weeks, it confirms that the time to achieve a benefit from RT may be more prolonged than the time achieve symptom relief in patients treated with RFA [5].

Conventional palliative external beam RT for painful metastases includes several different local and wide-field methods [7]. Unfortunately in about 60% of patients, this treatment is complicated by toxic effects, including nausea, vomiting, and diarrhoea.

Chemotherapy, by reducing the cancer volume, usually reduces pain in 20%-80% of patients.

A positive response often occurs within 2 weeks and can last for several months. Unfortunately, patients develop multidrug resistance, and recurrence of bone pain is common. Toxic effects, especially because of myelosuppression, are also common with chemotherapy for bone pain [8].

Up to 70% of patients with cancer report relief from bone pain after hormonal therapy or after single or multiagent chemotherapy [8]. However, hormonal therapy appears to be effective only in patients with breast or prostate cancer.

Radiopharmaceuticals, which have known benefit in patients with diffuse painful metastases involving bone, are not considered standard of care for patients with isolated, painful lesions.[4]

Surgery is usually reserved for patients with impending fracture or with spinal cord compression [6]. Analgesic medications are the first line of treatment for bone pain in cancer [9]. The World Health Organization [9] recommends a progressive 3-step approach starting with no steroidal anti-inflammatory drugs such as aspirin, ibuprofen, and naproxen to relieve mild to moderate pain. If pain persists or increases, step 2 adds a weak opioid such as codeine or hydrocodone. For persistent or moderate to severe pain, step 3 calls for more potent or higher doses of opioids such as morphine, hydromorphone, or fentanyl on a continuous or as-needed basis. Their efficacy may be improved by the concurrent administration of tricyclic antidepressants or phenothiazine. The use of many of these agents can be hindered by their substantial side effects, which often complicate the treatment of patients with cancer pain, including constipation, limitations in physical and mental status [9].

RFA represent a valid and potential alternative method for palliation of painful osteolytic metastases and many publications confirmed that RFA is a safe and effectiveness procedure in the treatment of localized, painful osteolytic metastases involving bone [4,5]. The mechanism of action responsible for decreased pain at the metastatic site after RFA includes: (a) physical destruction of adjacent sensory nerve fibres involving the periosteum and cortex of bone, inhibiting pain transmission; (b) mechanical decompression of cancer volume, decreasing stimulation of sensory nerve fibres; (c) destruction of tumour cells that produce nerve-stimulating cytokines (TNF α, interleukins, and others), which may sensitize nerve fibres and affect pain transmission; and (d) inhibition of osteoclast activity, which may cause pain [10].

In addition with RFA, other Authors [11-13] proposed the use of cementoplasty. The rational of this combined therapy is the stabilization of microfractures and reduction of mechanical forces, especially for larger tumours. In Toyota et al. [11] using RFA combined with cementoplasty for bulky tumours extending to extraosseus region obtain an analgesic reduction in only 41% of patients; probably the cause of this difference compared with RFA alone are due to the fact tumours’ size were relatively larger in this series. Percutaneous RFA and cement injection is widely used to treat painful metastasis involving bone but have relatively higher risk of complications. They are often associated with epidural extensions or posterior cortical disruption, witch may increase risk for leakage and subsequent compromise of thecal sac during cement delivery [11].

Schaefer et al [14] reported that RF heat ablation has proved to be an effective method for the treatment of skeletal disease; because of the coagulation necrosis produced by the RF heat ablation, a homogenous distribution of cement in the lesion was possible [14].

Georgy et al [15] have demonstrated that dissolution of tissue by plasma-mediated RFA rather than displacement to create a cavity before injecting bone cement permitted well-directed cement deposition into the compromised vertebral body, which may allow a safer procedure to be conducted in patients with advanced malignant vertebral compression fractures. Clinical benefits may include avoiding more extensive surgery and reducing the risk of complications associated with conventional bone cement injection procedures.

A tissue cavity can be achieved using other means, such as the kyphoplasty system. We believe that the advantage of using plasma-mediated RF based technology is the ability to create such a cavity using a tissue dissolution process, as opposed to simply displacing it. Moreover this technique provides the additional theoretic benefit of improving interdigitation of the cement, which normally be blocked by displaced tissue.

According with Georgy experience [15], we believe that this new technique present the inability to visualize the presumed cavity created by ablation; this was difficult to see in the post-procedure CT-scan examinations for two reasons. First because the cement was presumably deposited at the site of the create void and second because of the beam-hardening artefacts. The presence of gas in the needle tract stemming from tissue dissolution or the pattern of cement deposition inside the lytic lesion, represent two important indirect indicators of the tissue cavity created.

## Conclusion

Although larger series are needed to confirm our data, our experience suggest that plasma-mediated RFA assisted with cement injection is a new and potentially efficacy treatment for painful extra-vertebral bone metastases.

This technique may be safer than conventional cementoplasty of RFA associated to cementoplasty by providing a space for bone cement placement and plasma-mediated RFA could be more efficacious than non plasma-mediated because it limits the cement leakage damage to the nearby tissues.

Further clinical experience and prospective studies are needed to determine the long-term efficacy of combined RFA and percutaneous cementoplasty.

**Figure 2 (A) & (B).**
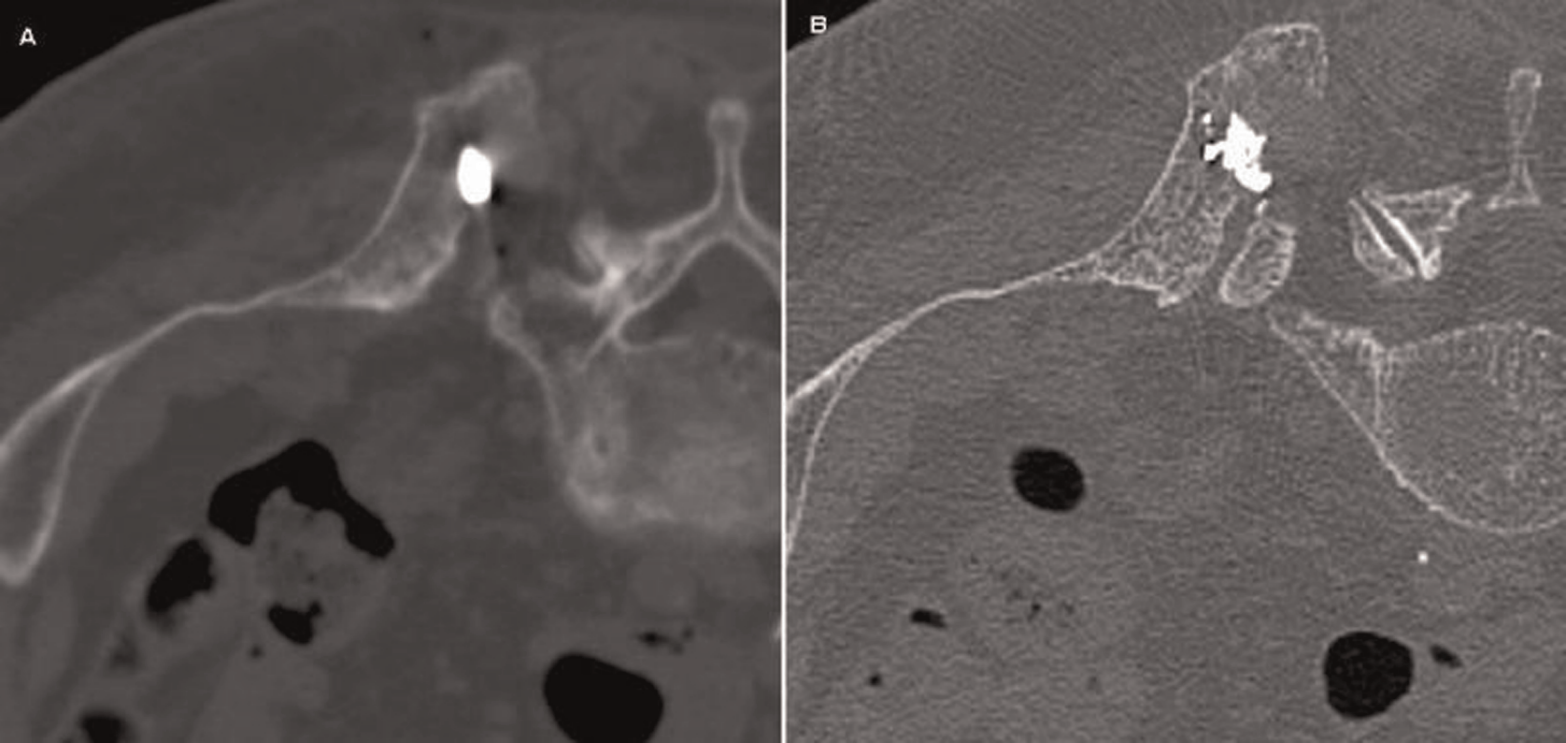
CT scan during the procedure. The electrode device is deployed inside the metastatic lesion and directed anteriorly through the malignant mass **(A)**; after completing the ablation a mixture of bone cement and sterile barium sulfate was injected into the ablated cavity **(B)**.

**Figure 3.**
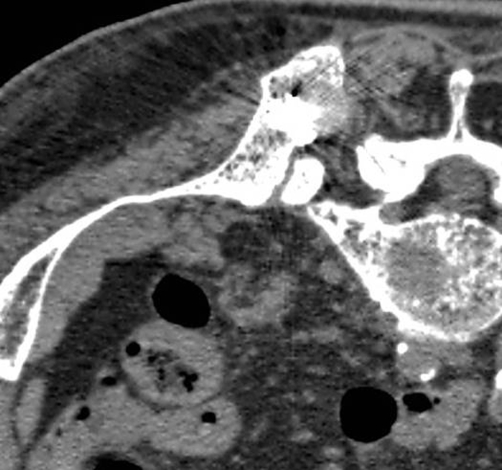
CT scans after the treatment. CT scans shows the distribution of cement inside the cavity created into the lesion.

## Abbreviations

BPI: Brief Pain Inventory
CT: Computer Tomography
RFA: Radio Frequency Ablation
RF: Radio Frequency
RT: Radiation Therapy
TNF: Tumour Necrosis Factor
VAS: Visual Analogue Scale
